# Health Literacy and Change in Health-Related Quality of Life in Dialysed Patients

**DOI:** 10.3390/ijerph19020620

**Published:** 2022-01-06

**Authors:** Ivana Skoumalova, Andrea Madarasova Geckova, Jaroslav Rosenberger, Maria Majernikova, Peter Kolarcik, Daniel Klein, Andrea F. de Winter, Jitse P. van Dijk, Sijmen A. Reijneveld

**Affiliations:** 1 Department of Health Psychology and Research Methodology, Faculty of Medicine, P. J. Safarik University, Trieda SNP 1, 040 11 Kosice, Slovakia; andrea.geckova@upjs.sk (A.M.G.); jaroslav.rosenberger@upjs.sk (J.R.); peter.kolarcik@upjs.sk (P.K.); 2 Graduate School Kosice Institute for Society and Health, Faculty of Medicine, P. J. Safarik University, Trieda SNP 1, 040 11 Kosice, Slovakia; a.f.de.winter@umcg.nl (A.F.d.W.); j.p.van.dijk@umcg.nl (J.P.v.D.); 3 Department of Community & Occupational Medicine, University Medical Center Groningen, University of Groningen, Antonius Deusinglaan 1, 9713 AV Groningen, The Netherlands; s.a.reijneveld@umcg.nl; 4 Olomouc University Social Health Institute, Palacky University, Univerzitní 22, 771 47 Olomouc, Czech Republic; 5 FMC-Dialysis Services Slovakia, Trieda SNP 1, 040 11 Kosice, Slovakia; mamajern@gmail.com; 6 II. Internal Clinic, Faculty of Medicine, P. J. Safarik University, Trieda SNP 1, 040 11 Kosice, Slovakia; 7 Institute of Mathematics, Faculty of Science, P. J. Safarik University, Jesenná 5, 040 01 Kosice, Slovakia; daniel.klein@upjs.sk

**Keywords:** health-related quality of life, health literacy, dialysed patients, change in quality of life

## Abstract

Health-related quality of life (HRQoL) is likely to deteriorate with the progression of chronic kidney disease (CKD). This change may be worsened by low health literacy (HL). We performed a longitudinal study at over 20 dialysis clinics in Slovakia (n = 413; mean age = 64.8 years; males = 58.4%). We assessed the association of three HL groups with a change in HRQoL over two years using binary logistic regression adjusted for type of vascular access, dialysis effectiveness, comorbidity, age and gender. We found that patients with low HL had poorer HRQoL at baseline in comparison to high-HL patients. We did not find significant associations of lower HL with the deterioration of mental or physical HRQoL after two years. In the adjusted model, patients with lower HL were not more likely to have deteriorated physical (low-HL patients: odds ratio/95% confidence interval: 0.99/0.53–1.84; moderate-HL patients: 0.97/0.55–1.73) or mental HRQoL (low-HL patients: 1.00/0.53–1.87; moderate-HL patients: 0.95/0.53–1.70) in comparison to high-HL patients. The HRQoL of lower-HL patients is worse at baseline but develops similarly to that of high-HL patients during dialysis treatment. Their relative HRQoL, thus, does not worsen further, but it does not improve either. Tailoring care to their needs may help to decrease the burden of low HL in dialysed patients.

## 1. Introduction

Chronic kidney disease (CKD) is a serious public health problem worldwide [1,2]. In the last 30 years, the global prevalence of CKD has increased by almost 30%, resulting in a prevalence of 9.1% in 2017 [3]; additionally, the global incidence of stage 5 CKD has increased rapidly [4]. CKD poses a great challenge for health policies and finances, especially regarding the treatment of stage 5 CKD, the final and most advanced stage when renal replacement therapy, such as dialysis (haemodialysis/peritoneal dialysis) or kidney transplantation, is required to keep patients alive. CKD is associated with increased morbidity and mortality [5], and with a higher burden observed in countries with a lower level of socioeconomic development and poorer access to healthcare [2]. It considerably affects patients’ lives due to its symptom burden, but also its treatment burden in advanced stages due to frequent dialysis, diet restrictions, changes in social functioning and employment, and the side effects of the dialysis itself, such as pain [6]. 

Health-related quality of life (HRQoL) is an important health outcome, especially in patients who live with a chronic condition. HRQoL in patients with CKD is lower than that of the general population [7], and it tends to decrease with the progression of the disease [8,9]. Impaired kidney function affects patients’ HRQoL by means of symptom burden, such as frailty, fatigue, pain, poorer physical functioning, weakness and cramps [10,11,12]. Many other factors are associated with HRQoL, such as the type of renal replacement therapy [13]; type of vascular access [14]; dialysis effectiveness [15]; comorbidity [16]; social support [17]; marital and socioeconomic status [18]; coping strategies [19]; and psychological well-being, e.g., depression and anxiety [20,21]. Additionally, poor HRQoL is associated with higher morbidity and increased risk of mortality in dialysed patients [22].

Health literacy (HL) is the ability to access, understand, appraise and apply health information to prevent illnesses and to maintain health during the course of life [23]. It is a multidimensional factor that covers several competencies related to searching for health information, self-management and cooperation with healthcare providers [24]. Low HL is common in dialysed patients [25,26] and is associated with poorer HRQoL [27,28,29]. All aforementioned studies reported significant associations of low HL with poorer mental and physical HRQoL in dialysed patients [27] and also in patients in earlier stages of the disease (3–5 stage) [28]. Our previous study [29] added to this evidence by providing detailed profiles of HRQoL in dialysed patients by level of HL.

HL may also contribute to a change in HRQoL [30] during treatment, but evidence on this association in dialysed patients is entirely lacking. There are a few studies on other chronic diseases focused on this association, such as in patients with type 2 diabetes [31] and asthma [32], which found a significant association of HL with the worsening of HRQoL. The aim of our study was to assess whether lower HL is associated with the worsening of HRQoL in a two-year follow-up in dialysed patients.

## 2. Materials and Methods

### 2.1. Sample and Procedure

We performed a follow-up study on a national sample of dialysed patients in Slovakia. We collected the baseline data from January to November 2018 and the follow-up data two years later within a network of 20 dialysis clinics. Dialysis treatment is reimbursed by the public health insurance system in Slovakia. At baseline, we included patients with a diagnosis of stage 5 CKD, aged over 18 years and undergoing dialysis for at least 90 days. We excluded patients with acute severe intercurrent illnesses and those who were not able to participate due to psychiatric diagnosis or an inability to speak Slovak, similar to other studies concerning CKD patients [25,26].

Data were obtained from patients using questionnaires, and from the medical database, i.e., the European Clinical Database, by extracting them (EuClid5; [33]). At baseline, the research assistant approached patients during their visit to the dialysis centre; after providing full information about the study, patients were asked to participate and signed informed consent. They then filled in the questionnaires using tablets. At the two-year follow-up, we approached patients in a similar way, and after being informed about the study, they again signed the informed consent and filled in online questionnaires. A unique personal identification code was also used to pair the follow-up data to ensure the full confidentiality of the provided data. The study was approved by the Ethics Committee of the Faculty of Medicine P. J. Safarik University (15N/2017) and the Ethics Committee of FMC-dialysis services.

### 2.2. Measures

Health-related quality of life was measured using the Kidney Disease Quality of Life-Short Form (KDQoL-SF 1.3) [34]. For the purpose of this study, we used only the second part of the questionnaire, the SF-36, which provides the generic core on HRQoL. We computed a physical component score (PCS) and a mental component score (MCS). The higher the scores of the PCS and MCS, the better the quality of life. We dichotomized this variable at baseline into lower (<40) and higher (≥40) PCS and MCS [35,36]. To obtain information on a change in HRQoL, we computed the mean difference between baseline and follow-up PCS and MCS data and dichotomized this variable as stable or improved vs. deteriorated HRQoL. Deteriorated HRQoL comprised the group whose scores decreased by 0.5 standard deviation (SD [37]) and more; improved HRQoL comprised an increase of 0.5 SD and more; the remaining data were labelled as stable. The latter two categories were combined. Patients who died during the follow-up period were included in the group of patients with deteriorated HRQoL.

HL was measured with the Health Literacy Questionnaire (Slovak version, [38]), a multidimensional tool for measuring HL [39]. It consists of nine HL domains. The higher the mean score in a particular domain, the higher the HL. We categorized this variable using hierarchical cluster analysis [40], which allowed us to obtain three groups of patients with different levels of HL considering all nine domains of HL. The procedure for handling this variable is described in a previous study [41]. Three HL groups were identified (low, moderate and high HL), and were used for the further analyses.

Sociodemographic data were measured using a questionnaire, and included age and gender.

The clinical measures obtained from the medical database included data on the age-adjusted Charlson Comorbidity index (CCI; the scores ranged from 0 to 33, with higher scores indicating higher comorbidity), type of vascular access (venous catheter vs. arteriovenous fistula) and haemodialysis effectiveness based on eKt/V with a cut-off of 1.4 (≤1.4 insufficient dialysis effectiveness vs. >1.4 sufficient dialysis effectiveness [15]). We used baseline clinical data in the further analyses.

### 2.3. Statistical Analyses

First, we assessed the sociodemographic characteristics of the sample and the prevalence for the three HL groups of the baseline clinical data and of the outcome variables (baseline HRQoL and change in HRQoL). Second, we assessed the association of the HL categories (low, moderate and high) with lower mental and physical HRQoL at baseline, using logistic regression analysis. Third, we assessed the associations of the three HL categories with deteriorated HRQoL, and also used logistic regression analysis. We adjusted the analyses for CCI (continuous-level variable), vascular access (dichotomised), dialysis effectiveness (dichotomised), age (continuous-level variable, centred age and age squared) and gender. For each outcome, we reported an odds ratio (OR) with the 95% confidence interval (CI). A *p*-value of <0.05 was assumed for statistical significance. We repeated the analysis excluding deceased patients (n = 89) as a form of sensitivity analysis. Statistical analyses were performed with SPSS v. 23.0 for Windows [42].

## 3. Results

### 3.1. Baseline and Follow-Up Characteristics

**Figure 1:**
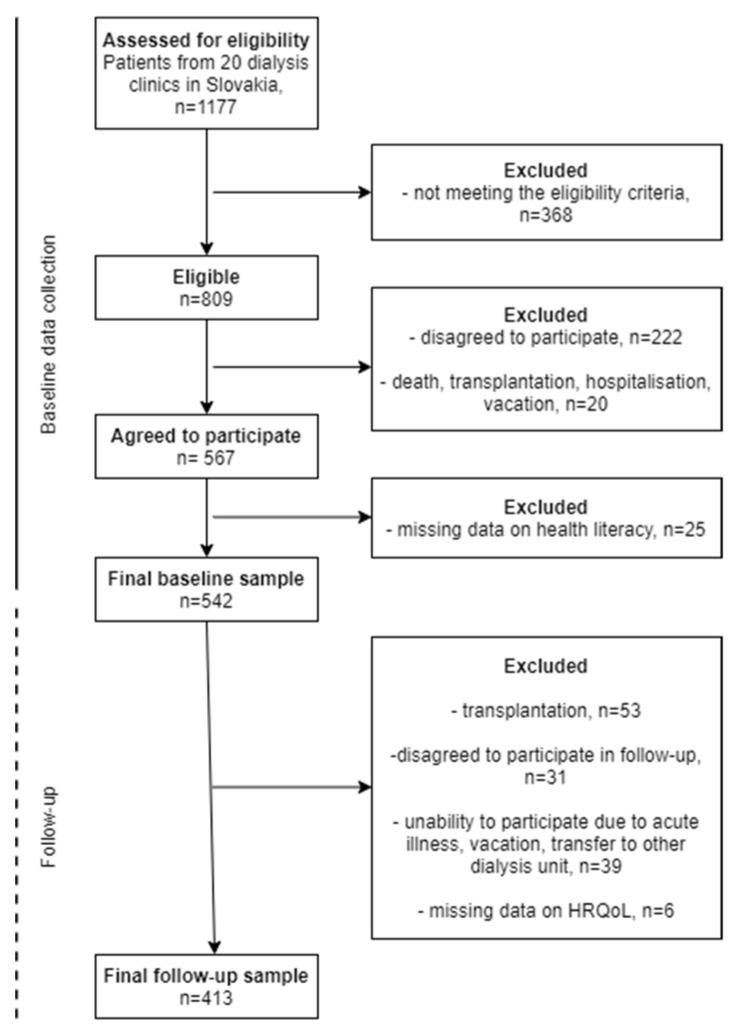
Flow chart of the sample.

We included 567 patients on maintenance dialysis. Follow-up information was obtained on 413 dialysed patients; 154 patients were lost to follow-up due to various reasons specified in the flow chart (Figure 1). The mean age of this sample at baseline was 64.8 years, and more than half were males (58.4%). Furthermore, 27% of the patients had a venous catheter; 20% of the patients had insufficient dialysis effectiveness. The mean CCI was 6.8. HL was low for 31.0%, moderate for 53.5% and high for 15.5%. At baseline, 71.7% of patients reported lower physical HRQoL, and 31.5% reported lower mental HRQoL. At follow-up, after two years, 49.4% patients had deteriorated physical HRQoL, and 40.9% had deteriorated mental HRQoL, including deceased patients (n = 89) (Table 1).

### 3.2. Association of Health Literacy with Baseline Health-Related Quality of Life

We found that patients in the low-HL group were more likely to have a worse physical HRQoL (odds ratio, OR: 2.59; 95% confidence interval, CI: 1.30–5.32) and mental HRQoL (OR 2.95; 95% CI: 1.39–6.23) than patients in the high-HL group. Patients in the moderate-HL group were more likely to have worse mental HRQoL (OR 2.16; 95% CI: 1.06–4.41). The findings are described in Table 2.

### 3.3. Health Literacy and Deterioration in Health-Related Quality of Life

We did not find a significant association of HL with deterioration in mental or physical HRQoL, either crude (model 1) or adjusted for confounders (model 2). In the adjusted model, patients with low HL were not more likely to have deteriorated physical HRQoL (OR 0.93; 95% CI: 0.49–1.75) or mental HRQoL (OR 0.88; 95% CI: 0.46–1.67) in comparison to high-HL patients. This also holds for patients with moderate HL—physical (OR 0.91; 95% CI: 0.51–1.62) and mental (OR 0.90; 95% CI: 0.50–1.63) HRQoL (Table 3). Repeating the analysis with the exclusion of deceased patients yielded similar findings (Table 4). 

## 4. Discussion

We explored the association of HL with changes in HRQoL during dialysis treatment in dialysed patients. Although we found that, at baseline, patients with lower HL had worse HRQoL than patients with higher HL, we found patients with lower HL not to be more likely to have deteriorated mental or physical HRQoL in a two-year follow-up period in comparison to patients with higher HL. To the best of our knowledge, this is the first study that provides evidence on the associations of HL with changes in HRQoL among dialysed patients.

We cannot compare our findings of similar changes in HRQoL for low-HL patients and for high-HL patients during dialysis treatment with previous research, as none is available. Two studies on other chronic conditions showed lower HL to be associated with a worsening of HRQoL. Sayah, Qui and Johnson [31] found that low HL was associated with worsening HRQoL in patients with type 2 diabetes, especially regarding its mental component. However, they used a different measurement tool to assess HL, the Brief Health Literacy Survey, which contains three screening questions regarding functional HL [43], whereas we used the much longer and multidimensional HLQ. Mancuso and Rincon [32] found that lower HL, also measured using a one-dimensional HL measuring tool, was associated with the worsening of the quality of life in asthma patients. However, this association attenuated after the inclusion of other independent variables (asthma knowledge and self-efficacy) in the analyses, implying the importance of other factors affecting patients’ outcomes, such as HRQoL.

The results of our study suggest that low HL does not add to a decrease in HRQoL in CKD patients when they are already in the stage 5 CKD and haemodialyzed. Taken in reverse, an interpretation of our results may also be that care does not sufficiently address their needs to help them reduce their gap in HRQoL compared to high-HL patients. Other factors, such as social support or the ability to cope with other diseases such as depression, may also play an important role in maintaining and improving patients’ HRQoL in this stage of the disease. This topic requires further study and more evidence.

### 4.1. Strengths and Limitations

The strength of our study lies in the large and nationally representative study sample of dialysed patients, the use of valid and reliable instruments to measure HL and HRQoL and the longitudinal design of the study. Moreover, this study is the first to assess the association of HL with longitudinal HRQoL in dialysed patients.

Our study also has some limitations. First, our use of self-reported questionnaires may have led to information bias and measurement error, which may be seen as a limitation in proper estimates. Another limitation may be due to the selection criteria, resulting in the potential exclusion of patients with the worst HL and probably the worst HRQoL. 

### 4.2. Implications

Our finding that low HL is associated with worse HRQoL in dialysed patients at baseline but is not associated with a change in mental or physical HRQoL during dialysis implies that patients with lower HL remain in a disadvantaged position in relation to their HRQoL. Therefore, more attention should be paid to patients with lower HL skills in order to support them to maintain their quality of life. Healthcare professionals should be able to identify HL limitations in patients, such as the inability to understand health information and instructions related to medication, self-care or self-management, or an inability to be actively engaged with healthcare providers, such as passivity and inability to ask questions regarding their health and symptoms. The additional training of medical students and health professionals may help to address this issue [44,45]. This finding also points to the need to focus on other important factors that may support improving HRQoL in dialysed patients, such as social support and better coping strategies. 

Future research should provide evidence on the effect of interventions aimed at increasing patients’ HL in order to identify whether such interventions may affect the HRQoL of dialysed patients in a positive way during their haemodialysis treatment, either in a clinically significant way, as assessed in this study, or as shown by any increase in mean HRQoL. Furthermore, it should focus on unveiling the pathways that lead from HL to HRQoL, e.g., by assessing the degree to which the association of HL with HRQoL at baseline is determined by other factors that mediate this relationship, such as additional clinical variables to those we assessed, such as anaemia or blood pressure. Such evidence may also provide further pathways for reducing the disadvantages of low-HL patients when undergoing dialysis treatment.

## 5. Conclusions

Lower HL is associated with worse HRQoL in dialysed patients, but HL is not associated with changes in HRQoL during dialysis treatment. More attention should be paid to patients with lower HL even in the early stages of CKD to avoid deterioration in HRQoL before the disease progresses to stage 5 CKD, where it is no longer related to HL skills. Moreover, improving their disadvantaged HRQoL position during dialysis treatment deserves the utmost attention.

## Figures and Tables

**Table 1:** Characteristics of the sample: gender; age; baseline clinical data; health literacy (HL); baseline health-related quality of life (HRQoL); and change in HRQoL, frequencies or means (n = 413, patients from 20 dialysis clinics in Slovakia).

Patients Characteristics	Total Sample n (%)
**Sociodemographic**	
Male gender	241 (58.4%)
Age (mean ± SD)	64.8 ± 13.7
**Baseline clinical data**	
Venous catheter	112 (27.3%)
Insufficient dialysis effectiveness	82 (20.1%)
Charlson comorbidity index (mean ± SD)	6.8 ± 2.9
**Health literacy**	
Low	128 (31.0%)
Moderate	221 (53.5%)
High	64 (15.5%)
**HRQoL at baseline ^1^**	
Lower physical HRQoL	296 (71.7%)
Lower mental HRQoL	130 (31.5%)
**Change in HRQoL ^2^**	
Deteriorated physical HRQoL	204 (49.4%)
Deteriorated mental HRQoL	169 (40.9%)

SD—standard deviation. ^1^ HRQoL at baseline was dichotomised as lower (<40) and higher (≥40) physical and mental HRQoL. The scores ranged from 0 to 100. In the table, we report the prevalence of lower physical and mental HRQoL in our sample. ^2^ Change in HRQoL was obtained by computing the mean difference between baseline and follow-up HRQoL; this was dichotomized as stable or improved (0) vs. deteriorated HRQoL (1). Deteriorated HRQoL comprised the group whose scores decreased by at least 0.5 standard deviation. In this table, we report the prevalence of deteriorated physical and mental HRQoL in our sample.

**Table 2:** Association of health literacy (HL) with lower health-related quality of life (HRQoL) at baseline, crude (model 1) and adjusted for Charlson comorbidity index, vascular access, dialysis effectiveness, age and gender (model 2): results of logistic regression analysis leading to odds ratios (OR) and 95% confidence intervals (CI), n = 413, patients from 20 dialysis clinics in Slovakia.

	Model 1 (Crude)		Model 2 (Adjusted)	
	Lower HRQoL at Baseline		Lower HRQoL at Baseline	
	physical	mental	physical	mental
	OR (95% CI)	OR (95% CI)	OR (95% CI)	OR (95% CI)
**Health literacy**				
Low	2.60 (1.33–5.10) **	2.98 (1.43–6.27) **	2.59 (1.30–5.32) **	2.95 (1.39–6.23) **
Moderate	1.32 (0.74–2.36)	2.23 (1.10–4.54) *	1.34 (0.72–2.48)	2.16 (1.06–4.41) *
High	Ref.	Ref.	Ref.	Ref.

* *p* < 0.05; ** *p* < 0.01; ref.: reference category.

**Table 3:** Association of health literacy (HL) with deteriorated health-related quality of life (HRQoL), crude (model 1) and adjusted for Charlson comorbidity index, vascular access, dialysis effectiveness, age and gender (model 2): results of logistic regression analysis leading to odds ratios (OR) and 95% confidence intervals (CI), n = 413, patients from 20 dialysis clinics in Slovakia.

	Model 1 (Crude)		Model 2 (Adjusted)	
	Deteriorated HRQoL		Deteriorated HRQoL	
	physical	mental	physical	mental
	OR (95% CI)	OR (95% CI)	OR (95% CI)	OR (95% CI)
**Health literacy**				
Low	1.13 (0.62–2.07)	1.07 (0.58–1.96)	0.93 (0.49–1.75)	0.88 (0.46–1.67)
Moderate	1.00 (0.57–1.74)	0.98 (0.56–1.74)	0.91 (0.51–1.62)	0.90 (0.50–1.63)
High	Ref.	Ref.	Ref.	Ref.

Ref.: reference category.

**Table 4:** Association of health literacy (HL) with deteriorated health-related quality of life (HRQoL), crude (model 1) and adjusted for Charlson comorbidity index, vascular access, dialysis effectiveness, age and gender (model 2): results of logistic regression analysis leading to odds ratios (OR) and 95% confidence intervals (CI), sensitivity analysis with exclusion of those who died; n = 324, patients from 20 dialysis clinics in Slovakia.

	Model 1 (Crude)		Model 2 (Adjusted)	
	Deteriorated HRQoL		Deteriorated HRQoL	
	physical	mental	physical	mental
	OR (95% CI)	OR (95% CI)	OR (95% CI)	OR (95% CI)
**Health literacy**				
Low	1.00 (0.49–2.07)	0.86 (0.38–1.94)	0.87 (0.41–1.85)	0.70 (0.30–1.64)
Moderate	1.12 (0.58–2.17)	1.15 (0.56–2.39)	1.02 (0.52–2.02)	1.07 (0.50–2.27)
High	Ref.	Ref.	Ref.	Ref.

Ref.: reference category.

## Data Availability

The data that support the findings of this study are available from the corresponding author upon reasonable request.

## References

[1] Jha V., Garcia-Garcia G., Iseki K., Li Z., Naicker S., Plattner B., Saran R., Wang A.Y., Yang C.W. (2013). Chronic kidney disease: Global dimension and perspectives. Lancet.

[2] Jha V., Modi G.K. (2018). Getting to know the enemy better-the global burden of chronic kidney disease. Kidney Int..

[3] GBD Chronic Kidney Disease Collaboration (2020). Global, regional, and national burden of chronic kidney disease, 1990–2017: A systematic analysis for the Global Burden of Disease Study 2017. Lancet.

[4] Cockwell P., Fisher L.A. (2020). The global burden of chronic kidney disease. Lancet.

[5] Tonelli M., Wiebe N., Culleton B., House A., Rabbat C., Fok M., McAlister F., Garg A.X. (2006). Chronic kidney disease and mortality risk: A systematic review. J. Am. Soc. Nephrol..

[6] Samoudi A.F., Marzouq M.K., Samara A.M., Zyoud S.H., Al-Jabi S.W. (2021). The impact of pain on the quality of life of patients with end-stage renal disease undergoing hemodialysis: A multicenter cross-sectional study from Palestine. Health Qual. Life Outcomes.

[7] Perlman R.L., Finkelstein F.O., Liu L., Roys E., Kiser M., Eisele G., Burrows-Hudson S., Messana J.M., Levin N., Rajagopalan S. (2005). Quality of life in chronic kidney disease (CKD): A cross-sectional analysis in the Renal Research Institute-CKD study. Am. J. Kidney Dis..

[8] Pagels A.A., Söderkvist B.K., Medin C., Hylander B., Heiwe S. (2012). Health-related quality of life in different stages of chronic kidney disease and at initiation of dialysis treatment. Health Qual. Life Outcomes.

[9] Mujais S.K., Story K., Brouillette J., Takano T., Soroka S., Franek C., Mendelssohn D., Finkelstein F.O. (2009). Health related quality of life in CKD Patients: Correlates and evolution over time. Clin. J. Am. Soc. Nephrol..

[10] Nixon A.C., Wilkinson T.J., Young H.M.L., Taal M.W., Pendleton N., Mitra S., Brady M.E., Dhaygude A.P., Smith A.C. (2020). Symptom-burden in people living with frailty and chronic kidney disease. BMC Nephrol..

[11] Li H., Xie L., Yang J., Pang X. (2018). Symptom burden amongst patients su_ering from end-stage renal disease and receiving dialysis: A literature review. Int. J. Nurs. Sci..

[12] Abdel-Kader K., Unruh M.L., Weisbord S.D. (2009). Symptom burden, depression, and quality of life in chronic and end-stage kidney disease. Clin. J. Am. Soc. Nephrol..

[13] Chuasuwan A., Pooripussarakul S., Thakkinstian A., Ingsathit A., Pattanaprateep O. (2020). Comparisons of quality of life between patients underwent peritoneal dialysis and hemodialysis: A systematic review and metaanalysis. Health Qual. Life Outcomes.

[14] Afsar B., Elsurer R., Covic A., Kanbay M. (2012). Vascular access type, health-related quality of life, and depression in hemodialysis patients: A preliminary report. J. Vasc. Access.

[15] Levin A., Stevens P.E., Bilous R.W., Coresh J., De Francisco A.L.M., De Jong P.E., Griffith K.E., Hemmelgarn B.R., Iseki K., Lamb E.J. (2013). Kidney disease: Improving global outcomes (KDIGO) CKD work group. KDIGO 2012 clinical practice guideline for the evaluation and management of chronic kidney disease. Kidney Int. Suppl..

[16] Cha J., Han D. (2020). Health-Related Quality of Life Based on Comorbidities Among Patients with End-Stage Renal Disease. Osong Public Health Res. Perspect..

[17] Untas A., Thumma J., Rascle N., Rayner H., Mapes D., Lopes A.A., Fukuhara S., Akizawa T., Morgenstern H., Robinson B.M. (2011). The associations of social support and other psychosocial factors with mortality and quality of lie in the Dialysis outcomes and Practice Patterns Study. Clin. J. Am. Soc. Nephrol..

[18] Molsted S., Wendelboe S., Flege M.M., Eidemak I. (2021). The impact of marital and socioeconomic status on quality of life and physical activity in patients with chronic kidney disease. Int. Urol. Nephrol..

[19] Schick-Makaroff K., Molzahn A.E., Kalfoss M. (2018). Symptoms, Coping, and Quality of Life of People with Chronic Kidney Disease. Nephrol. Nurs. J..

[20] Kaltsouda A., Skapinakis P., Damigos D., Ikonomou M., Kalaitzidis R., Mavreas V., Siamopoulos K.C. (2011). Defensive coping and health-related quality of life in chronic kidney disease: A cross-sectional study. BMC Nephrol..

[21] Tong A., Sainsbury P., Chadban S., Walker R.G., Harris D.C., Carter S.M., Hall B., Hawley C., Craig J.C. (2009). Patients’ experiences and perspectives of living with CKD. Am. J. Kidney Dis..

[22] Mapes D.L., Lopes A.A., Satayathum S., McCullough K.P., Goodkin D.A., Locatelli F., Fukuhara S., Young E.W., Kurokawa K., Saito A. (2003). Health-related quality of life as a predictor of mortality and hospitalization: The Dialysis Outcomes and Practice Patterns Study (DOPPS). Kidney Int..

[23] Sørensen K., Van den Broucke S., Fullam J., Doyle G., Pelikan J., Slonska Z., Brand H., (HLS-EU) Consortium Health Literacy Project European (2012). Health literacy and public health: A systematic review and integration of definitions and models. BMC Public Health.

[24] Batterham R.W., Hawkins M., Collins P.A., Buchbinder R., Osborne R.H. (2016). Health literacy: Applying current concepts to improve health services and reduce health inequalities. Public Health.

[25] Green J.A., Mor M.K., Shields A.M., Sevick M.A., Palevsky P.M., Fine M.J., Arnold R.M., Weisbord S.D. (2011). Prevalence and demographic and clinical associations of health literacy in patients on maintenance hemodialysis. Clin. J. Am. Soc. Nephrol..

[26] Taylor D.M., Fraser S.D.S., Bradley J.A., Bradley C., Draper H., Metcalfe W., Oniscu G.C., Tomson C.R.V., Ravanan R., Roderick P.J. (2017). A Systematic Review of the Prevalence and Associations of Limited Health Literacy in CKD. Clin. J. Am. Soc. Nephrol..

[27] Dodson S., Osicka T., Huang L., McMahon L.P., Roberts M.A. (2016). Multifaceted Assessment of Health Literacy in People Receiving Dialysis: Associations With Psychological Stress and Quality of Life. J. Health Commun..

[28] Stømer E.U., Wahl K.A., Gøransson G.L., Urstad H.K. (2020). Health literacy in kidney disease: Associations with quality of life and adherence. J. Ren. Care.

[29] Skoumalova I., Madarasova Geckova A., Rosenberger J., Majernikova M., Kolarcik P., Klein D., de Winter A.F., van Dijk J.P., Reijneveld S.A. (2021). Health-related quality of life profiles in dialyzed patients with varying health literacy. A cross-sectional study on Slovak haemodialyzed population. Int. J. Public Health.

[30] Paasche-Orlow M.K., Wolf M.S. (2007). The causal pathways linking health literacy to health outcomes. Am. J. Health Behav..

[31] Sayah F.A., Qiu W., Johnson J.A. (2016). Health literacy and health-related quality of life in adults with type 2 diabetes: A longitudinal study. Qual. Life Res..

[32] Mancuso C.A., Rincon M. (2006). Impact of health literacy on longitudinal asthma outcomes. J. Gen. Intern. Med..

[33] Marcelli D., Kirchgessner J., Amato C., Steil H., Mitteregger A., Moscardò V., Carioni C., Orlandini G., Gatti E. (2001). EuCliD (European Clinical Database): A database comparing different realities. J. Nephrol..

[34] Hays R.D., Kallich J.D., Mapes D.L., Coons S.J., Carter W.B. (1994). Development of the kidney disease quality of life (KDQOL) instrument. Qual. Life Res..

[35] Cioncoloni D., Innocenti I., Bartalini S., Santarnecchi E., Rossi S., Rossi A., Ulivelli M. (2014). Individual factors enhance poor health-related quality of life outcome in multiple sclerosis patients. Significance of predictive determinants. J. Neurol. Sci..

[36] Nagasawa H., Sugita I., Tachi T., Esaki H., Yoshida A., Kanematsu Y., Noguchi Y., Kobayashi Y., Ichikawa E., Tsuchiya T. (2018). The Relationship Between Dialysis Patients’ Quality of Life and Caregivers’ Quality of Life. Front. Pharmacol..

[37] Norman G.R., Sloan J.A., Wyrwich K.W. (2003). Interpretation of changes in health-related quality of life: The remarkable universality of half a standard deviation. Med. Care.

[38] Kolarcik P., Cepova E., Madarasova Geckova A., Elsworth G.R., Batterham R.W., Osborne R.H. (2017). Structural properties and psychometric improvements of the Health Literacy Questionnaire in a Slovak population. Int. J. Public Health.

[39] Osborne R.H., Batterham R.W., Elsworth G.R., Hawkins M., Buchbinder R. (2013). The grounded psychometric development and initial validation of the Health Literacy Questionnaire (HLQ). BMC Public Health.

[40] Ward J.H. (1963). Hierarchical Grouping to Optimize an Objective Function. J. Am. Stat. Assoc..

[41] Skoumalova I., Madarasova Geckova A., Rosenberger J., Majernikova M., Kolarcik P., Klein D., Winter A.F., van Dijk J.P., Reijneveld S.A. (2020). Does Depression and Anxiety Mediate the Relation between Limited Health Literacy and Diet Non-Adherence?. Int. J. Environ. Res. Public Health.

[42] IBM Corp. (2015). IBM SPSS Statistics for Windows.

[43] Chew L.D., Griffin J.M., Partin M.R., Noorbaloochi S., Grill J.P., Snyder A., Vanryn M. (2008). Validation of screening questions for limited health literacy in a large VA outpatient population. J. Gen. Intern. Med..

[44] Kaper M.S., Reijneveld S.A., van Es F.D., de Zeeuw J., Almansa J., Koot J.A.R., de Winter A.F. (2019). Effectiveness of a comprehensive health literacy consultation skills training for undergraduate medical students: A randomized controlled trial. Int. J. Environ. Res. Public Health.

[45] Kaper M.S., de Winter A.F., Bevilacqua R., Giammarchi C., McCusker A., Sixsmith J., Koot J.A.R., Reijneveld S.A. (2020). Positive outcomes of a comprehensive health literacy communication training for health professionals in three European countries: A multi-centre pre-post intervention study. Int. J. Environ. Res. Public Health.

